# Biochemical evidence for the tyrosine involvement in cationic intermediate stabilization in mouse β-carotene 15, 15'-monooxygenase

**DOI:** 10.1186/1471-2091-10-31

**Published:** 2009-12-14

**Authors:** Eugenia Poliakov, Susan Gentleman, Preethi Chander, Francis X Cunningham, Bella L Grigorenko, Alexander V Nemuhin, T Michael Redmond

**Affiliations:** 1 National Eye Institute, NIH, Bethesda, MD 20892-0608, USA; 2 Department of Cell Biology and Molecular Genetics, University of Maryland, College Park, Maryland 20742, USA; 3 Chemistry Department, MV Lomonosov Moscow State University, Moscow, Russia

## Abstract

**Background:**

β-carotene 15,15'-monooxygenase (BCMO1) catalyzes the crucial first step in vitamin A biosynthesis in animals. We wished to explore the possibility that a carbocation intermediate is formed during the cleavage reaction of BCMO1, as is seen for many isoprenoid biosynthesis enzymes, and to determine which residues in the substrate binding cleft are necessary for catalytic and substrate binding activity. To test this hypothesis, we replaced substrate cleft aromatic and acidic residues by site-directed mutagenesis. Enzymatic activity was measured *in vitro *using His-tag purified proteins and *in vivo *in a β-carotene-accumulating *E. coli *system.

**Results:**

Our assays show that mutation of either Y235 or Y326 to leucine (no cation-π stabilization) significantly impairs the catalytic activity of the enzyme. Moreover, mutation of Y326 to glutamine (predicted to destabilize a putative carbocation) almost eliminates activity (9.3% of wt activity). However, replacement of these same tyrosines with phenylalanine or tryptophan does not significantly impair activity, indicating that aromaticity at these residues is crucial. Mutations of two other aromatic residues in the binding cleft of BCMO1, F51 and W454, to either another aromatic residue or to leucine do not influence the catalytic activity of the enzyme. Our *ab initio *model of BCMO1 with β-carotene mounted supports a mechanism involving cation-π stabilization by Y235 and Y326.

**Conclusions:**

Our data are consistent with the formation of a substrate carbocation intermediate and cation-π stabilization of this intermediate by two aromatic residues in the substrate-binding cleft of BCMO1.

## Background

Carotenoid polyene oxygenases are a diverse superfamily whose members play a crucial role in carotenoid/retinoid metabolism, producing, among other actions, key hormones such as abscisic acid in plants [1] and vitamin A in animals [2]. Three mammalian family members with very different functions have been described: 1) β-carotene-15,15'-monooxygenase (BCMO1)^1 ^is an enzyme which symmetrically cleaves β-carotene to produce two molecules of all-*trans *retinal [3], and is the initial step in biosynthesis of vitamin A in animals [2,4]; 2) β-carotene-9',10'-monooxygenase (BCMO2) is believed to cleave β-carotene asymmetrically to produce 10'-apocarotenal and β-ionone [5]; and 3) RPE65 is an isomer(ohydrol)ase in the visual cycle, converting all-*trans *retinyl palmitate to 11-*cis *retinol [6-8]. In BCMO1 knockout mice, disruption in BCMO1 leads to hypercarotenemia on a provitamin A-rich, vitamin A-deficient diet and also to changes in lipid metabolism and susceptibility to diet-induced obesity [9]. Pathological effects of mutations in BCMO1 [10] have also been described in humans. Therefore, the catalytic mechanism for this enzyme is of particular interest.

Based on the solved apocarotenoid-15,15'-oxygenase (ACO) crystal structure, all these iron (II)-dependent oxygenases are predicted to share a common seven bladed β-propeller structure [11]. In ACO, Fe(II) is coordinated in a near-perfect octahedral arrangement by four histidines. The fifth position in the crystal structure is occupied by a water molecule, and dioxygen is proposed to complete the coordination geometry at the sixth position. Presumably, all members of this family, including BCMO1, share this iron center arrangement. Recently, we validated the crystal structure predictions by showing that mutations of the invariant histidines H172A, H237A, H308A and H514A which are paralogs of the iron-coordinating histidines in ACO abolished BCMO1 activity. Although still controversial, the central cleavage of β-carotene by BCMO1 is currently believed to follow a monooxygenase mechanism [12]. On the other hand, it has unequivocally been shown that the plant family member CCD1 is a dioxygenase [13]. Experiments by Olson *et al*. with crude enzyme preparations led the authors to believe that BCMO1 is also a dioxygenase [14,15]. However, the substituted bis(1-alkylimidazol-2-yl)propionate mononuclear iron complexes, which closely resemble ACO iron coordination, efficiently catalyze both olefin epoxidation (monoxygenation products) and *cis*-dihydroxylation (dioxygenation products) [16], suggesting that both mechanisms are plausible for the various carotenoid oxygenases.

The process of monooxygenation is suggested to involve reductive oxygen activation at metal complexes followed by oxygen transfer to the substrate [17]. In the ACO crystal structure, all-*trans *carotenoid substrate was modeled in a 13,14-13',14'-di-*cis *conformation [11]. The formation of cation intermediates in the process of *trans*- to *cis*-isomerization is well documented for electrochemical, ferric oxide and iodine oxidation of carotenoids [18-20]. Also, cationic intermediates were predicted in an *ab initio *study of lycopene isomerization, providing a quantum mechanical explanation for the antioxidant properties of carotenoids [21]. Recently it has been demonstrated that a carotenoid cation-radical is formed in the photosynthetic light harvesting system [22,23]. In addition, the analysis of the ACO oxidative cleavage mechanism by the quantum chemical density field theory method predicted formation of a substrate radical cation [24]. A cation-radical is formed by one electron oxidation of β-carotene; a second one-electron oxidation leads to formation of a dication β-carotene. Electron paramagnetic resonance (EPR) and electrochemical studies of carotenoid oxidation led to a proposed equilibrium between radical cation, dication and the original carotenoid. Moreover, careful analysis of the electrochemical behavior of β-carotene in methylene chloride demonstrated that two electrons are transferred in the first oxidation step, and thus formation of dications occurs at lower oxidation potential [20]. Simultaneous electrochemical/EPR experiments showed subsequent decay of the β-carotene dication to a monocation as a result of the proton transfer [20]. On the other hand, carbocation could be produced by protonating the β-carotene carbon-carbon double bond chain. Diamagnetic carbocations exhibit considerable stability at room temperature due to charge delocalization [25]. A large body of published work points to stabilization of carbocations by anionic and aromatic residues in active sites of polyene chain synthesizing enzymes [26-28]. Recently, an intramolecular proton-transfer mechanism has been implicated in a carbocationic mechanism of farnesyl pyrophosphate synthase and aristolochene synthase [29,30]. Therefore we have investigated the question of involvement of carbocation intermediates during the enzymatic cleavage of β-carotene.

In the present report, we have analyzed the role of conserved catalytic site aromatic residues in the catalytic mechanism of BCMO1, using site-directed mutagenesis to create a set of mutant enzymes and characterizing these mutants using *in vitro *and *in vivo *assays.

## Results

### Modeling of mouse BCMO1 on the ACO template and identification of conserved aromatics in the substrate binding cleft

**Figure 1:**
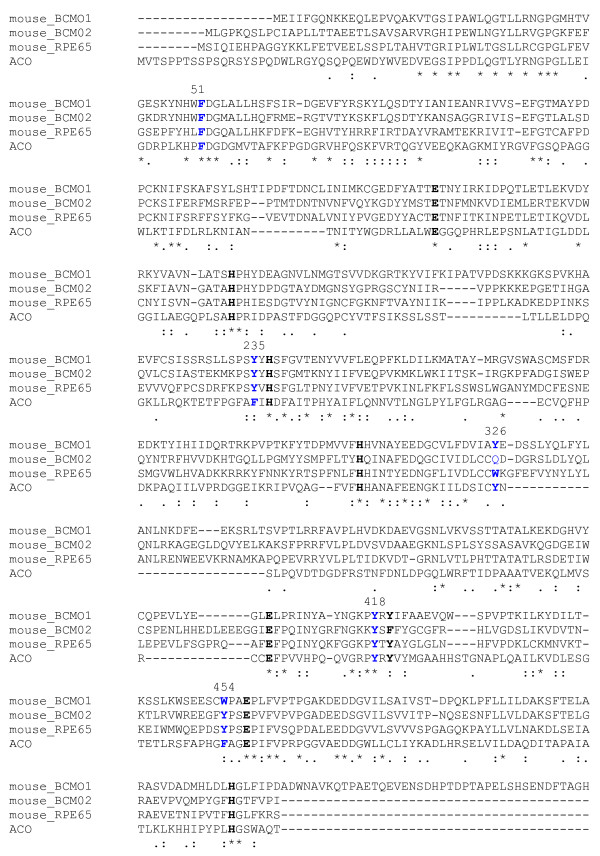
**ClustalW protein sequence alignment of murine polyene oxygenase family members with ACO**. Conserved iron-coordinating residues are in bold black, conserved tyrosines in bold blue and the acidic residues of the signature sequence in bold red. Nonconserved aromatics studied are in blue. The last 4 amino acids of mouse BCMO1 were removed. GenBank™/EBI accession numbers are as follows: mouse BCMO1, AF271298; mouse BCMO2, AJ290392; mouse RPE65, NM_029987; Synechocystis LSD (ACO), D90914. Alignment coding: * identity, : strong similarity, weak similarity.

**Figure 2:**
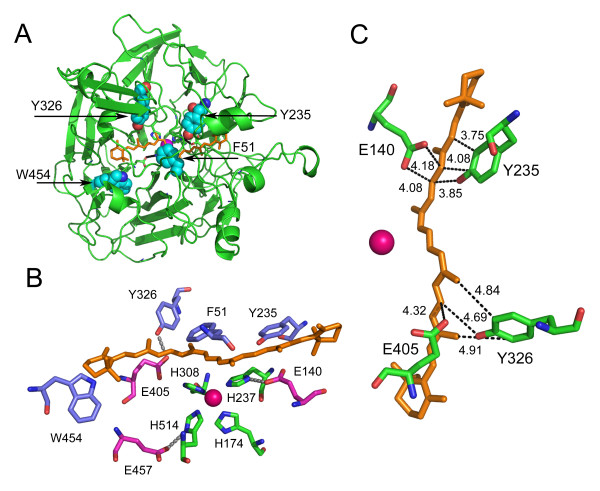
**PyMol modeling of BCMO1 on the ACO template**. **A**) Aromatic residues in the binding cleft of BCMO1. Selected aromatic residues of BCMO1 are plotted to equivalent positions on the carbon backbone of the ACO crystal structure [11] by using the PyMol program. Blue, aromatics side chains; red, backbone oxygen; blue, backbone nitrogen; dark pink, iron center; gold, beta-carotene. **B**) Visualization of the aromatic and acidic BCMO1 residues of the catalytic site on the ACO crystal structure. Aromatic residues of the substrate binding region modeled on the ACO template (PyMol) are shown. Violet, aromatic side chains; pink, acidic side chains; green, iron-coordinating histidines side chains; red, backbone oxygen; blue, backbone nitrogen; red-pink, iron center; gold, beta-carotene. **C**) Model of the tyrosines and acidic residues in the substrate binding cleft of BCMO1. PyMol model of BCMO1 (tyrosines and "fixing" glutamates in radius <5 Å from β-carotene are shown) on apocarotenoid oxygenase (ACO) template with *ab initio *mounted β-carotene. A possible hydrogen bond is predicted between Y326/E405.

Preliminary ClustalW 1.74 alignment of BCMO1 and ACO was optimized for conserved histidines and acidic residues. This alignment was iteratively presented to the Swiss Model Repository with manual adjustment after each cycle for the final complete model. The alignment shows several conserved aromatic residues, particularly in regions near the Fe-coordinating histidines (Fig. 1). Both these enzymes cleave carotenoids at the 15-15' position. Using this alignment to model the structure of BCMO1 (Fig. 2A) we found four aromatics within the substrate cleft paralogous to aromatics in ACO: F51 (ACO = F69) in the highly conserved FDG motif above the Fe, Y235 (ACO = F236) above E140 fixing the second coordinating histidine (H237), Y326 (ACO = Y322) above E405 fixing the third coordinating histidine (H308), and W454 (ACO = W423) near E457 fixing the fourth coordinating histidine (H514) (Fig. 2B). F51, Y235 and Y326 are predicted to be less than 5 Å from the polyene chain of the substrate, and W454 is similarly close to one of the β-ionone rings of the substrate (Fig. 2C).

To determine the role of these aromatic residues and the nearby acidic residues in the catalytic mechanism of BCMO1, we constructed a set of mutants in which each of these aromatic residues was replaced with leucine or an alternative aromatic. Y326 was also exchanged to the paralogous glutamine, as in BCMO2. In previous work, we had determined the effect of mutating the conserved acidic residues to alanine [31].

### Analysis of aromatics in the substrate binding cleft *in vivo*

Since mutation of the crucial residues in the enzyme leads to significant reduction in the activity of BCMO1, the sensitivity of *in vitro *enzymatic assay is not sufficient for analysis of these residues[32]. Therefore, we made use of β-carotene-accumulating *E.coli/*pAC-BETA [33] as an *in vivo *system for measurement of enzymatic activity.

Substrate for BCMO1 (β-carotene) is constitutively synthesized and accumulated in this specially designed *E.coli *strain. After we allowed sufficient time for β-carotene to be accumulated in the cells, we induced synthesis of BCMO1 and measured the amount of beta-carotene left in induced and uninduced cells. As previously described [31], uninduced pBAD/BCMO1 constructs are functionally equivalent to the irrelevant pBad/LacZ construct (in which there is no carotene cleavage) for use as negative controls of BCMO1 activity.

**Figure 3:**
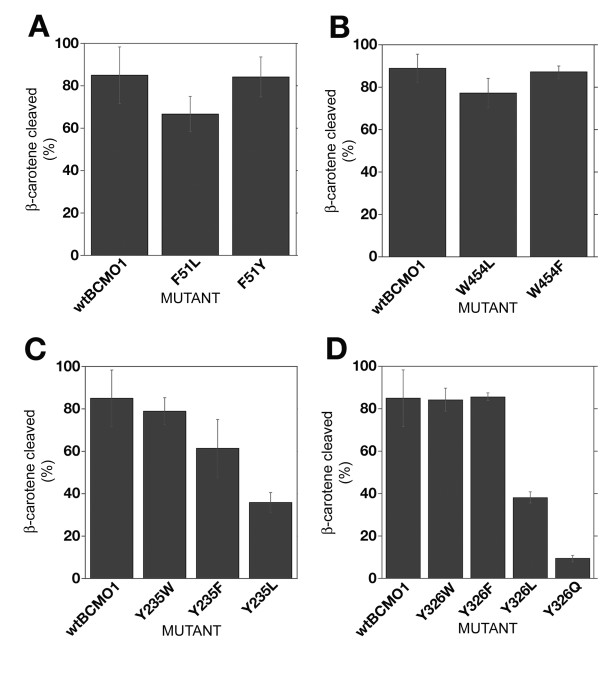
**B-carotene cleavage activity of mutants of aromatic residues located in binding cleft of BCMO1**. **A**) Mutations of F51. **B**) Mutations of W454. **C**) Mutations of Y235. **D**) Mutations of Y326. Mid-log phase cultures of β-carotene producing *E. coli *transformed with pBAD/BCMO1 (+ control) or pBAD/mutant BCMO1 constructs were split in half; one half was induced with 0.02% L-arabinose, while the other was not. Both cultures were grown for 3 hours at 30°C in the dark and harvested. β-carotene was extracted and analyzed by RP-HPLC. To account for differences in the production of β-carotene in various cell cultures prior to induction we expressed β-carotene cleavage activity as (1-n) × 100%, where n is a ratio of β-carotene extracted from induced culture to β-carotene extracted from uninduced cell culture, normalized to protein concentration (+/-SE, N = 3).

Mutation of either F51 or W454 to leucine had little effect on the activity of BCMO1 and mutation of F51 to tyrosine or W454 to phenylalanine had no effect in this assay (Fig. 3A, B). However, a more significant effect was seen with the two tyrosines, Y235 and Y326. In the case of Y235, practically no loss of activity was observed with the Y235W mutant, moderate loss of activity with the Y235F mutant, and greater loss with the Y235L mutant (Fig. 3C). Y235 is located adjacent to the E140 "fixing" glutamate in the substrate binding cleft (~3.8 A), for which the E140A mutation has been shown to reduce activity to about 50% relative to WT [31]. Thus, these two residues could play a role in the catalytic mechanism of the enzyme. Analysis of the double mutants for Y235F/E140A and Y235L/E140A revealed residual activity (bleaching) only for Y235F/E140A mutant 20 h post-induction (data not shown). These results support the idea that this pair is not involved in proton transfer for formation of carbocations but may point to compensating mechanisms in substrate stabilization. As we noted above, Y326 is located on our model less than 5 Å from β-carotene and 2.73 Å from E405. To address the function of Y326 in the BCMO1 catalytic mechanism, we constructed a set of mutants: Y326W (as in RPE65), Y326F, Y326L and Y326Q (as in BCMO2). Catalytic activity of Y326W (84.2 ± 5.4) is comparable to that of the wild type BCMO1 (82.4 ± 5.6) in the accumulative *in vivo *system (Fig. 3D) and Y326F is also active. Mutation to the nonaromatic amino acid leucine, Y326L, resulted in a drop in catalytic activity (38.2 ± 2.6). The most significant decrease in catalytic activity was observed by introduction of the partially positively charged amino acid glutamine in place of tyrosine: Y326Q (9.3 ± 1.5). (Fig. 3D). Our previous studies had demonstrated that, of the three "fixing" glutamates, only E405 is indispensable for enzyme activity as the E405A mutant has no activity, although the protein is expressed [31]. Thus, compensating mechanisms are unlikely for this pair of residues. These data collectively demonstrate that aromaticity of the residue is important for the enzyme activity in both the Y235 and Y326 positions but not in the F51 or W454 positions.

### Expression of mutant constructs

**Figure 4:**
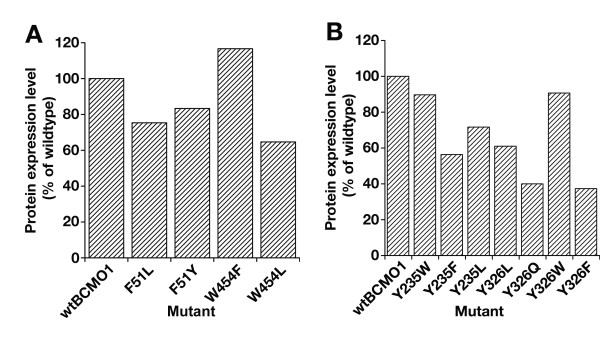
**Expression of mutant BCMO1 constructs in transformed cell lines**. **A**) Quantification of expression of F51 and W454 mutants. **B**) Quantification of expression of Y235 and Y326 mutants. The cell extracts were probed with a monoclonal anti-His antibody. The intensity of each mutant BCMO1 band was quantified by densitometry and normalized to intensity of recombinant BCMO1 (0.1 μg) synthesized by Roche RTS cell-free transcription/translation on each gel.

To examine the correlation of enzyme activity with *in vivo *protein expression levels for the various mutants, immunoblots of extracts from 10 cell lines transformed with mutant BCMO1 constructs were analyzed by His-tag and rabbit polyclonal BCMO1 specific antibodies (Fig. 4A, B). Endogeneous EF-Tu bacterial protein (MW = 49,541Da) was used as a loading control. When compared with wildtype, the drastic decrease in mutant enzyme activity observed *in vivo *correlated with somewhat lowered expression levels, but these changes in activity could not be explained solely by a change in expression levels.

### *In vitro *analysis of enzymatic activity of BCMO1 tyrosines

In our *in vitro *assay system we determined K_m _and V_max _for Y326W, and two mutants on the propeller axis, Y418F and Y420F. Tyrosines 418 and 420 are fairly well conserved among mammal family members and are located far from the catalytic site of the enzyme. In Y326W, we observed a 4-fold decrease in V_max _and 4-fold increase in K_m_, whereas Y420 F did not show significant changes in kinetics and Y418F shows 3-fold decrease in V_max _and only 2-fold increase in K_m _(Table 1). We were unable to determine kinetics for the Y235F mutant due to insufficient sensitivity of the *in vitro *assay used in our studies here, although at the highest substrate concentration technically possible in this assay system, a low level of activity was observed (data not shown). This suggests a probable increase in K_m _for the Y235F mutant. Also, the substantial increase in K_m _observed only in the substrate cleft residues supports the hypothesis that they play a role in substrate stabilization. Neighboring to Y235, the Y236F mutant showed also about a 3 fold increase in K_m _and a more pronounced 5 fold decrease in V_max_. Expression of the Y236L mutant was almost abolished (5% of wt BCMO1) in our system, and this fact points to a possible structural role for this residue.

**Table 1:** Biochemical Characteristics of wt BCMO1 and enzymatically active mutants^a^.

Enzyme	K_m _(μM)	V_max_ (pmol/μg*hour)
BCMO1wt	4.7	121.2

Y420F	3.6	109.9

Y418F	9.8	39.7

Y326W	20.1^#^	31.8

Y236F	15.4^#^	22.7

^a^K_m _[μM] and V_max _[pmol retinal/hour* μg enzyme] were calculated from Hanes [s/v *vs *s] plots. Calculation of enzymatic activity was based on the production of retinal from β-carotene. Data was calculated based on average substrate curve for each protein.

^# ^These data are K_m _apparent values due to limitations of β-carotene solubility in reaction mixture (the highest concentration of β-carotene is 42 μM).

### Diphenylamine is an inhibitor of BCMO1

**Figure 5:**
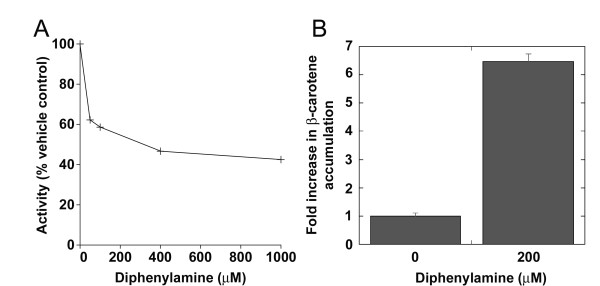
**Inhibition of BCMO1 by diphenylamine**. **A**) Inhibition of BCMO1 by diphenylamine *in vitro*. Diphenylamine (50-1000 μM) was added from a 20 mM stock in DMSO to an *in vitro *reaction mixture prior to addition of substrate. The final concentration of DMSO in all samples was 5%. The enzyme was preincubated with the inhibitor for 30 min at 4°C in the standard reaction buffer and was then added to 20 μM β-carotene and activity determined as described in Methods. A representative experiment is shown. **B**) Inhibition of BCMO1 by diphenylamine *in *vivo. E. *coli *cultures transformed with both the plasmid for β-carotene synthesis and the BCMO1 plasmid were grown overnight (12-16 hr) to allow accumulation of β-carotene. At this point, the inhibitor diphenylamine (at a final concentration of 200 μM from a 200 mM stock solution in MeOH) was added to prevent further synthesis of carotenoids [38]. The cultures were shaken for 2 hours, split in half, and one half was induced by the addition of arabinose (0.02% final concentration). Both cultures were shaken for an additional 7 hours and analyzed by the *in vivo *assay as described in Methods (N = 3).

Many enzymes involving a transient carbocation mechanism are effectively inhibited by positively charged amine derivatives [34-36]. The inhibitory potency of these amine derivatives is linked to their ability to be protonated at physiological pH (noncompetitive inhibition) [34] or to their similarity to transition state carbocation intermediates (competitive inhibition). To further elucidate the mechanism of action of BCMO1, we examined the effects of various amine inhibitors of carotenoid synthesis such as nicotine (gamma and beta cyclases) [34], galactosamine (xanthophyll epoxidase) [36] and diphenylamine (phytoene desaturase and β-carotene ketolase) [37] on enzyme activity. Neither nicotine nor galactosamine significantly inhibited BCMO1 (data not shown); however, diphenylamine inhibited BCMO1 at sub-millimolar concentrations *in vitro *(Fig. 5A) Diphenylamine, which inhibits carotenoid synthesis completely at 200 μM concentration [38] also impaired β-carotene degradation significantly our *in *vivo assay in β-carotene-accumulating *E.coli *expressing BCMO1 (Fig. 5B).

## Discussion

In multiple protein sequence alignment of vertebrate carotenoid oxygenase family member sequences (Fig. 1), we found that all members conserved aromatics in positions comparable to F51, Y235, and W454. With respect to Y326 in BCMO1, Y was conserved in other BCMO1 orthologs, W was the paralogous residue in RPE65, but aromatic resides were not found in BCMO2 sequences at this position. It should be noted that BCMO1 and RPE65 both act at the 15 carbon position, whereas BCMO2 is thought to cleave carotenoids asymmetrically. The Swiss-PDB model of BCMO1 built on the ACO template predicts that F51, Y235, Y326 and W454 are aromatics located in the carotenoid binding cleft. The possibility that some or all of these residues participate in catalytically important functions was thus an important consideration. Tyrosines are known to play a key role in coupled proton-shuttling in a large number of metalloenzymes [39,40], and, additionally, can directly participate in electron transfer by forming tyrosyl radicals [41,42]. They may also play a structural role by participating in the architecture of the enzyme active site, or play a role in the correct positioning of the substrate and cofactor [43,44] and are known to take part in interaction with substrate and/or cofactors and determine the substrate specificity in a variety of enzymes [44-47]. Finally, tyrosine and other aromatic amino acids are implicated in cation-π interactions stabilizing carbocation intermediates generated during enzymatic reactions [27,48,49].

Our results indicate that mutations of Y235 and Y326 to leucine significantly impair catalytic activity of BCMO1. We found that the aromaticity of amino acids Y235 and Y326 are important for enzymatic activity, whereas mutation of F51 or W454 to leucine had little effect. Mutation of either of these two tyrosines to other aromatic residues (phenylalanine or tryptophan) does not affect enzyme activity by more than 10-20% relative to wild type, but replacement to non-aromatic residues (leucine or glutamine) leads to significant loss of enzymatic activity. The studies presented here support a mechanism implicating cation-π stabilization of a putative carbocation intermediate. We can exclude the possibility of radical formation by these tyrosines in BCMO1 catalysis, as the tyrosine to leucine mutants, Y235L and Y326L, although reduced in activity (sometimes significantly), exhibited some level of residual activity. Additionally, EPR analyses of BCMO1 failed to detect tyrosyl radical formation (Poliakov and Krishna, unpublished results). However, this finding does not exclude the possibility of other functional roles for these residues. The Swiss-PDB model of BCMO1 built on the ACO template predicts close localization of Y235-E140 and Y326-E405 residues with possible hydrogen bonding for the latter pair (~2.73 Å) (Fig. 2B). However, our mutagenesis data suggest that these hydrogen bonds cannot be crucial for the catalytic mechanism of BCMO1; moreover, simultaneous replacement of Y235F/E140A does not eliminate activity.

From our data it is reasonable to propose that aromaticity of the two tyrosines Y235 and Y326 plays an important role in the catalytic mechanism of BCMO1. To explore this possibility, we mounted an *ab initio *model of β-carotene onto the BCMO1 structural model by thermodynamic optimization. It was observed that tyrosines Y235 and Y326 fix the position of the substrate on two sides of the 15-15' double bond of the substrate (Fig. 2C). The predicted hydrophobic interactions with substrate are in accordance with our catalytic activity data for Y235F, Y235W and Y235L, and Y326W, Y326F and Y326L mutants. Consequently, we can speculate that in the case of a mechanism involving one-electron oxidation by dioxygen bound to the ferrous ion and the subsequent radical-cation formation [24], or two one-electron oxidation steps and dication formation [20], the aromatic residues (Y235, Y326) and glutamates (E140, E405) stabilize a cationic intermediate with charge delocalization along the polyene chain. On the other hand, we could not rule out the possibility of protonation of a double bond and formation of a carbocation as in the case of isopentyl diphosphate:dimethylallyl diphosphate isomerase [48]. Distances between these tyrosines and β-carotene chain in our model are less that 5 Å which is in good agreement with cation-π stabilization (Fig. 2C) [50]. The occurrence of such stabilization was previously observed in squalene-hopene cyclase and oxidosqualene-lanosterol cyclase [26,27]. In the polycyclization cascade of squalene and oxidosqualene, aspartates initiate the cyclization process by protonation of the terminal double bond and stabilize the first carbocation intermediate [28]. The π-electrons of several aromatic residues stabilize cationic intermediates in other enzymes [49,51,52]. The cation intermediate would explain the observation of transient substrate isomerization in the binding cleft of ACO [11]. Transient carbocations are formed in many steps of sterol and carotenoid synthesis, and various positively charged amino derivatives could noncompetitively inhibit these enzymes. Of the three inhibitors tested here, diphenylamine, an oxidation inhibitor, was the only one to have a substantial inhibitory effect on BCMO1 activity. Diphenylamine is reported to inhibit electron transfer in photosynthetic membranes [53] and carotenogenesis by inhibiting carotenoid ketolase and phytoene desaturase. However, this is in line with the common mechanism of inhibition by diphenylamine of iron oxygenases and is not necessarily conclusive for carbocation formation.

As expected, replacement of Y326 by tryptophan, which has the highest π-binding energy of the aromatic series [54], did not negatively effect activity *in vivo*. The altered biochemical parameters (especially the higher K_m_) for the Y326W mutant could be explained by steric hindrance. Also, it is important to note that the *in vivo *assay shows the cumulative effect of mutation and thus cannot estimate the catalytic efficiency of the enzyme. Replacement of tyrosine by glutamine (a partially positively charged residue)[55] would be expected to destabilize a cation, and therefore, effectively inhibit catalytic activity of BCMO1. The results presented here correlate with these hypotheses. Productive replacement of Y326 by tryptophan, such as found at the paralogous residue W331 in RPE65 [56] suggests that BCMO1 and RPE65 may share a similar catalytic mechanism. In fact, the formation of a carbocation intermediate had been proposed in the catalytic mechanism of retinoid isomerization in the RPE [57,58] prior to the identification of RPE65 as the isomerase [6-8].

## Conclusions

We show that aromaticity of the substrate binding cleft aromatic residues Y235 and Y326 is important for the activity of recombinant mouse BCMO1. The enzymatic activity changes follows the trend Y235W>Y235F>Y235L and Y326W~Y326F>Y326L>Y326Q which agrees very well with the π-binding energies of these amino acids. Based on the mutational data and structure modeling we propose that cation-π interactions between Y235 and Y326 and β-carotene cation intermediate play an important role in the mechanism of BCMO1. We also show that replacing F51 and W454, two other substrate binding cleft aromatic residues, does not affect BCMO1 enzyme activity. Insight into the mechanism of BCMO1 would help to understand carotenoid levels and metabolism in humans [59].

## Methods

### Site-directed mutagenesis and expression of the mouse BCMO1, and mutants

A panel of mutant mouse BCMO1 proteins was made using a previously described pBAD/BCMO1 construct containing the BCMO1 open-reading frame as template [4]. Site-directed mutagenesis of these proteins was done with the QuikChange XL site-directed mutagenesis kit (Stratagene, CA). The validity of all point mutations and the integrity of the open reading frames were verified by sequencing.

Wild-type BCMO1 and single point mutants containing a polyHis-tag fusion were expressed in a carotenoid-accumulating strain of *E.coli *or in TOP10 strain of *E.coli *as described previously [31].

### *In vivo *assay of enzymatic activity of the mouse BCMO1 and mutants

pBAD/BCMO1, pBAD/mutant BCMO1 or pBAD/LacZ expression constructs were transformed into competent cells prepared as described [60] from a strain of *E.coli *transformed with the vector pAC-BETA that produces and accumulates β-carotene [33]. Overnight cultures of *E.coli*/pAC-BETA with constructs were grown in LB broth supplemented with 100 μg/mL ampicillin and 30 μg/mL chloramphenicol at 30°C. One ml of the overnight culture was used to inoculate 50 mL of LB broth supplemented with the same antibiotics. The culture was allowed to grow at 37°C to mid-log phase (OD_600_~0.6) and was then split; one half was induced with 0.02% (w/v) of arabinose while the other was not induced. Both cultures were grown for another 3 hours at 30°C, optical density measured, and cells were collected by centrifugation at 5,000 g for 20 min. Pellets were frozen in dry ice and stored at -70°C. To quantify the activity of the various constructs, carotenoids were extracted from frozen bacterial cultures. Small aliquots (2 mL) were frozen for protein determinations and western blotting. The pellet was resuspended in acetone with 2.7% formaldehyde and 3% methanol (1 mL) containing β-8'-apo-carotenal (30 μM) as an internal standard. Cells were resuspended by sonication and vortexing and then were divided into three equal aliquots and each was centrifuged for 2 min at 16,060 g. Supernatants were collected, and carotenoids were extracted from each pellet with 2 sequential aliquots of 300 μL acetone with rigorous vortexing. Supernatants and acetone extracts were combined and filtered, and then 100 μL was injected onto a reverse phase HPLC (RP-HPLC) column (see below). The amount of β-carotene was quantified as the area of peak normalized to the area of the β-8'-apo-carotenal internal standard. Samples were assayed in triplicate from induced and uninduced cultures and the activity expressed as a (1-n) × 100%, where n is a ratio of β-carotene extracted from induced culture to β-carotene extracted from uninduced cell culture, normalized to protein concentration (+/-SE, N = 3).

### Immunoblotting

Mid-log phase cultures of *E.coli *were induced with 0.02% L-arabinose, grown for 3 hours at 30°C in the dark and harvested. The cell cultures (2 mL, OD~1.7), were resuspended in 200 μL of 50 mM Tris-HCl (pH 8), 300 mM NaCl solution with Complete Mini EDTA-free protease inhibitors (Roche, 1 tab/10 mL) and homogenized using the FastPrep device (QBiogen) according to the manufacturer's recommendations. Due to the restricted amount of available protein, equivalent protein from 3 extracts of a mutant was pooled for each mutant sample. 25 μg of total lysate protein was loaded per lane on the gel. The blots were probed with monoclonal anti-His antibodies (Roche) diluted 1:1,000 in 5% BSA in IBS for 1 hour, and after 3 washes with 1xIBS with secondary Goat Anti-mouse IgG-AP conjugate (Novagen) for 1 hour and developed colorimetrically with BCIP/NBP substrate (KPL). For quantification of expression, the cell extracts were probed with a polyclonal rabbit anti-BCMO1 peptide antibody (1:1,000 in 5% BSA in IBS) and then with the secondary goat anti-rabbit IgG-HRP conjugate (Novagen) and the ECL development (Amersham). The intensity of each mutant BCMO1 band was quantified by densitometry and normalized to the intensity of recombinant BCMO1 (0.1 μg) synthesized in the Roche RTS cell-free transcription/translation system as a standard on each gel.

### *In vitro *enzymatic activity assay

The *in vitro *assays for BCMO1 were performed following the method of Lindqvist and Andersson [32] with small modifications described previously [31]. Typically, reactions were run in glass vials under reduced light in a volume of 200 μL in 100 mM Tricine-KOH (pH 8.0) buffer supplemented with 10 μM FeSO_4_, 5 mM Tris(2-carboxyethyl)phosphine hydroxychloride (TCEP), 1% (w/v) 1-S-octyl-β-d-thioglucopyranoside (OTG) and 10-20% (v/v) glycerol. The enzyme concentration varied between 100 and 1600 ng/reaction, as determined by direct ELISA[31]. The concentration of β-carotene in the reaction mixture ranged from 0.1 to 42 μM from a stock solution of 90 pmol/μL β-carotene (the solubility of β-carotene is a problem at higher concentrations). The substrate was solubilized in hexane with 50 μL of 4% OTG in a glass vial, and the solvent was evaporated under argon. The assay buffer with substrate/detergent was preincubated at 37°C for 5 min, the enzyme in glycerol added, and the reaction incubated at 37°C in water bath for 1 hour. To analyze reaction products, we used a protocol modified from During *et al *[61]. 50 μL of 37% (v/v) formaldehyde was added to stop the reaction, and the incubation was continued for 10 min at 37°C. Then, 500 μL of acetonitrile was added, the solution vortexed and put on ice at 4°C for 5 min. The upper acetonitrile phase was collected and 100 μL injected and analyzed by RP-HPLC.

### HPLC analysis of carotenoids and all-*trans *retinal

β-carotene, all-*trans *retinal and β-apo-8'-carotenal (internal standard for *in vivo *assays) were separated on a 4.6 × 250 mm 3 μm YMC C30 carotenoid column Waters, Inc.) with a flow rate of 1 ml/min and simultaneous UV detection at 451 nm and 383 nm (Agilent 1100 HPLC), as described previously with small modifications [31]. Briefly, the initial conditions consisted of 60:40 acetonitrile/0.015 M NH_4_OAc held for 5 min, and followed by a linear gradient to 50:50 isopropanol/acetonitrile in 10 min. This was held for an additional 10 min, and then followed by a linear gradient to 80:20 isopropanol/acetonitrile in 5 minutes and held for an additional 2 minutes before returning to initial conditions.

β-carotene and all-*trans *retinal were quantified from their peak area using standard curves obtained with 2-220 ng and 0.5-20 ng of material, respectively, in 60/40 acetonitrile:lysis buffer.

### Tertiary structure modeling

Tertiary structure modeling was done using SWISSMODEL (version 36.0003). This protein structure homology-modeling server was accessed *via *the ExPASy web server and/or locally from the program DeepView-Swiss-PDB Viewer [62]. The template for modeling BCMO1 was the structure of apocarotenal oxygenase (ACO) from *Synechocystis *[11].

To mount the β-carotene molecule to the BCMO1 Swiss-Pdb model the following simulation protocol was applied. The DS ViewerPro 6.0 (Accelrys Software Inc.) was used for visualization and initial operation with the structures. Four histidine residues (His172, His234, His308 and His514) are predicted to belong to the coordination shell of Fe^2+^. This set of ligands was augmented by a water molecule and the dioxygen molecule to form the six-fold coordination shell of iron. The carotene molecule was then mounted manually by building its polyene chain inside the available space and optimizing temporary geometry configurations by using the "Clean structure" procedure of DS ViewerPro 6.0. Finally, an arrangement of the carotene species inside the protein binding pocket was optimized by using the quantum mechanical - molecular mechanical method [63].

### Statistical analysis

Calculations of mean, variance and standard errors were done using the Data Analysis Pack in Microsoft Office Excel program.

## Abbreviations

The abbreviations used are: ACO: apocarotenal oxygenase; BCMO1: β-carotene 15:15'-monooxygenase; BCMO2: β-carotene 9',10'-monooxygenase; BSA: bovine serum albumin; DEA: diethylamine; EDTA: ethylenediaminetetraacetic acid; ELISA: enzyme-linked immunosorbent assay; EPR: electron paramagnetic resonance; IBS: imidazole-buffered saline; OTG: 1-S-octyl-β-d-thioglucopyranoside; RP-HPLC: reverse phase high performance liquid chromatography; TCEP: Tris(2-carboxyethyl)phosphine hydrochloride.

## Authors' contributions

EP carried out all mutagenesis experiments and *in vitro *and *ex vivo *enzymatic assays and wrote the paper. SG ran multiple sequence alignments and developed BCMO1 Swiss-View Pro model based on the conserved motifs with ACO template, and helped write the paper. FC developed *ex vivo *assays for carotenoid-accumulating *E.coli *in the presence of carotenoid synthesis inhibitors. BG and AN performed quantum mechanical - molecular mechanical optimization of BCMO1 tertiary structure with modeled β-carotene. PC analyzed possible structure changes in the mutant enzymes in PyMOL Molecular Viewer. TMR supervised the project and helped write the paper. All the authors have read and approved the final version of the manuscript.
